# Additional Gastrectomy after Endoscopic Submucosal Dissection for Early Gastric Cancer Patients with Comorbidities

**DOI:** 10.1155/2012/379210

**Published:** 2012-05-08

**Authors:** Naohiko Koide, Daisuke Takeuchi, Akira Suzuki, Satoshi Ishizone, Shinichi Miyagawa

**Affiliations:** Department of Surgery, Shinshu University School of Medicine, Matsumoto 390-8621, Japan

## Abstract

*Purpose*. We investigated the clinicopathologic features of early gastric cancer (EGC) patients who have undergone additional gastrectomy after endoscopic submucosal dissection (ESD) because of their comorbidities. *Methods*. Eighteen (7.1%) of 252 GC patients were gastrectomized after prior ESD. Reasons for further surgery, preoperative and postoperative problems, and the clinical outcome were determined. *Results*. The 18 patients had submucosal EGC and several co-morbidities. Other primary cancers were observed in 8 (44.4%). Histories of major abdominal operations were observed in 6 (33.3%). Fourteen patients (77.8%) hoped for endoscopic treatment. Due to additional gastrectomy, residual cancer was suspected in 10, and node metastasis was suspected in 11. A cancer remnant was histologically observed in one. Node metastasis was detected in 3 (16.7%). Small EGC was newly detected in 4. Consequently, additional gastrectomy was necessary for the one third. No patient showed GC recurrence. However, 9 (50%) had new diseases, and 4 (22.2%) died of other diseases. The overall survival after surgery in these patients with additional gastrectomy was poorer than those with routine gastrectomy for submucosal EGC (*P* = 0.0087). *Conclusions*. Additional gastrectomy was safely performed in EGC patients with co-morbidities. However, some issues, including presence of node metastasis and other death after surgery, remain.

## 1. Introduction

Endoscopic treatments, including endoscopic mucosal resection (EMR) and endoscopic submucosal dissection (ESD), for early gastric cancer (EGC) have markedly progressed and are widely accepted [1–3]. EMR can be safely performed for EGC conditioned by differentiated mucosal EGC smaller than 20 mm in diameter [4], and the general indications were proposed by the Japanese Gastric Cancer Association. Recently, ESD is frequently used for the treatment of EGC because ESD has the advantage of curability on achieving successful en-block resection [1–3]. Furthermore, extended indications of EMR/ESD for EGC have been clinicopathologically investigated [5–8]. The expanded criteria of EMR/ESD for EGC are still controversial [9], and the expanded criteria need to be confirmed yet. Most mucosal EGC showed low-level lymph node metastasis while approximately 15–20% of submucosal EGC showed node metastasis [10]. There are cases that underwent additional gastrectomy after EMR/ESD, because cancer invades the submucosal layer of the stomach with/without lymphovascular invasion histopathologically detected in specimens obtained by EMR/ESD. Regarding criteria for EGC treatment, patients sometimes hope for endoscopic treatment, as it is less invasive compared to surgery. Haruma et al. [11] reported that endoscopic therapy in patients who are inoperable or have a high surgical risk appears effective. There have recently been reports regarding additional treatments, including gastrectomy, after incomplete EMR/ESD [12, 13]. Patients with gastrectomy after prior EMR/ESD may have several problems regarding the surgical management and long-term outcome. Especially, the clinicopathologic issues, excluding the histopathologic features of gastric cancer, in gastrectomized patients after prior ESD have not yet been investigated.

We retrospectively investigated the clinicopathologic issues concerning gastrectomized patients after ESD for EGC, and the reasons for ESD and further surgery for EGC, and pre- and postoperative problems were determined from a surgical viewpoint.

## 2. Patients and Methods

### 2.1. Patients

We retrospectively analyzed our database of all GC patients who underwent gastrectomy. A total of 252 patients with GC were newly diagnosed and surgically treated between 2004 and 2008 at Shinshu University Hospital. Eighteen patients (15 men and 3 women, 7.1%: Group A) were additionally gastrecomized after ESD for EGC. Sixty-four patients (49 men and 15 women, 25.4%: Group B) were routinely and electively gastrectomized for EGC with submucosal invasion.

### 2.2. Clinicopathologic Issues

The following parameters were investigated: (1) problems before treatments, (a) comorbidities, (b) multiple cancers of other organs, and (c) history of major abdominal surgery and invasive treatment for other diseases; (2) reason for treatment of EGC, (a) reason for receiving ESD, and (b) reason for additional gastrectomy (pathologic findings of ESD); (3) pathologic findings after additional gastrectomy; (4) postoperative complications; (5) clinical outcome after additional gastrectomy. Finally the clinicopathologic features in Group A were compared with those of Group B.

### 2.3. Endoscopic Treatment

ESD for EGC was performed by several gastrointestinal endoscopists in Shinshu University Hospital and other hospitals in Nagano Prefecture. One hundred and fifty-three patients received endoscopic treatments in the same period, and 15 (9.8%) of the 153 patients with additional gastrectomy received prior endoscopic treatments at our hospital. The remaining 3 visited from other hospitals for endoscopic treatment of EGC in our hospital. ESD for EGC was carried out employing a few methods using hook and flex knives or an insulation-tipped diathermic knife. Usually, mucosal EGC was indicated for ESD and en-block removal. The removed tissues were formalin fixed and paraffin embedded. The obtained sections were histopathologically examined and were routinely evaluated according to the Japanese Classification of Gastric Carcinoma established by the Japanese Gastric Cancer Association [14].

### 2.4. Gastrectomy after ESD

When the histopathologic findings after ESD showed submucosal invasion with/without lymphatic or venous invasions, additional gastrectomy was considered. Gastrectomy with regional node dissection for EGC with/without prior ESD was performed employing an open or laparoscopy-assisted approach. When EGC was located in the lower and middle thirds of the stomach, distal gastrectomy was performed. When EGC was located in the upper third of the stomach, total or proximal gastrectomy was performed. Regional nodes were routinely dissected using the procedure of D1 (with no. 7) or (with no. 7, 8a, and 11p) in patients with EGC. In advanced GC, D2-node dissection was usually performed. These resected specimens and lymph nodes were examined according to the routine histopathologic procedures for diagnosis and staging. The clinicopathologic features of GC were described according to the Japanese Classification of Gastric Carcinoma [14]. All 18 patients with additional gastrectomy were performed D1-lymphadenectomy: 14 with node dissection surrounding left gastric arteries and 4 with additional dissection of the nodes surrounding the common hepatic and splenic and celiac arteries.

### 2.5. Clinical Outcome after Surgery

For 5 years after surgery, these patients were followed in the outpatient clinic of Shinshu University Hospital in order to check for recurrence/metastasis of the tumors by esophagogastroduodenoscopy every year and computed tomography (CT) of the abdomen and chest every 6 months and/or annually. Death caused by recurrence/metastasis of GC or by other diseases after surgery was investigated.

### 2.6. Statistical Analysis

Data are shown as the prevalence, or mean and ordinal data were compared by the Mann-Whitney *U* and Chi-square or Fisher's exact probability test. Five-year survival rates after surgery were calculated employing the Kaplan-Meier method. *P* < 0.05 was considered significant.

## 3. Results

### 3.1. Problems before Treatments

**Comorbidities**

All 18 patients had one or more comorbidities before treatments for EGC (Table 1). Additionally, two of 4 patients with diabetes mellitus had not yet been treated, and insulin treatment was started for surgery. One with chronic obstructive pulmonary disease and one with idiopathic interstitial pneumonia routinely underwent home-oxygenic treatment. Two patients with dermatomyositis were treated with predonisolone long term.

**Multiple Cancers in Other Organs**

Other primary cancers were observed in 8 patients (44.4%: Table 2). Nine antecedent cancers in other organs were observed in 7 patients, while one synchronous cancer was observed in the liver. In these patients, 6 tumors had undergone surgical treatment.

**History of Major Surgery and Invasive Treatment**

Histories of major abdominal operations were observed in 6 patients (33.3%). Gastrectomy had been carried out for previous EGC in one patient. Colectomy for advanced colon cancer had been conducted in 2 patients. Oophorectomy for ovarian cancer and benign ovarian tumor had been performed in one each. Hysterectomy for uterine cancer had been performed in one patient. Hepatectomy for hepatocellular carcinoma and the extirpation of retroperitoneal liposarcoma had been performed in one. A history of invasive treatment/operation for other disorders was observed in 3 patients (16.7%): surgical clipping of cerebral aneurysm, coronary artery bypass grafting, or percutaneous coronary intervention for ischemic heart disease. These invasive treatments with/without surgery were performed in 9 patients (50.0%).

### 3.2. Reason for Treatment of EGC

**Reason for Receiving ESD**

Regarding the patients' opinions, 14 (77.8%) of the 18 patients hoped for ESD treatment for EGC. They gave the following reasons: their advanced age, comorbidities, history of abdominal surgery, and social convenience. Regarding the doctors' opinions, EGC was suspected as submucosal cancer, but diagnostic treatment was conducted in 4 patients (22.2%) by ESD.

**Reason for Additional Gastrectomy (Pathologic Findings of ESD)**

Twenty-three EGC were removed by ESD in the 18 patients; double lesions patients were removed in 5 patients (27.8%). Cut-end margins of 11 lesions in 10 patients (55.6%) were positive: the positive lateral margin in 3 patients (16.7%) and the positive vertical margin in 8 patients (44.4%). In one patient, both margins were positive. Submucosal invasion was demonstrated in 19 lesions of the 18 patients. Submucosal invasion less than 500 *μ*m from the muscularis mucosa (sm1) was shown in 5 lesions of 4 patients (22.2%), and submucosal invasion deeper than 500 *μ*m from the muscularis mucosa (sm2) in 14 lesions of 14 patients (77.8%). Histological vessel invasions, including lymphatic and/or venous invasions, were shown in 11 lesions: 2 lesions with sm1 and 9 lesions with sm2. Based on the histopathologic findings of EGC specimens after ESD, residual cancer was suspected in 10 patients, and node metastasis was suspected in 11 patients because of presence of histologic lymphatic and/or venous invasion. The remaining 2 showed a regional node swelling (over 10 mm in diameter) on abdominal CT in the followup after ESD. Finally, the 18 patients underwent additional gastrectomy.

### 3.3. Pathologic Findings after Surgery

In primary lesions, cancer remnant was observed in only one patient, while no remnant was observed in 17 patients (94.4%). New cancer was detected in the resected stomach after gastrectomy in 4 patients (22.2%); 2 of the 4 patients had a third cancer. These lesions were mucosal and less than 10 mm in diameter; majority of them shows 2–4 mm in size. Node metastasis was observed in 3 patients (16.7%). Two cases pathologically showed a metastatic node, and one had 4 metastatic node. The 2 cases with preoperatively suspected node metastasis on CT revealed no metastasis histologically. In addition, *Helicobacter pylori* was observed in 17 (94.4%), but the presence was same between the patients with single and multiple gastric cancer in Group A.

### 3.4. Postoperative Complications

Postoperative complications were observed in 9 patients (50.0%) in Group A. Liver dysfunction was observed in 3 patients; one of them showed massive ascites, approximately 1,000-mL drainage, every day after surgery. Infection of the central catheter was observed in 2 patients with total gastrectomy. Postoperative delirium was observed in 2 patients. Other postoperative complications were acute pancreatitis, pericarditis with heart failure, angina pectoris, and atrial fibrillation with tachycardia. These complications were conservatively treated, and consequently improved. In Group B, no case with anastomotic leakage was also observed. There was no difference of postoperative morbidity between Group A and Group B. No postoperative mortality was observed. In addition, there was no difference in operative blood loss and operating time between the two groups (Table 3).

### 3.5. Clinical Outcome after Surgery

In the followup, there was no case of additional gastrectomy with cancer recurrence/metastasis. New disorders after surgery were observed in 9 patients (50.0%). Mental/neurological diseases, including depression and dementia, were observed in 4 patients over 75 years old. Other primary cancers were subsequently observed in 2 patients (11.1%): hepatocellular carcinoma 26 months after and lung cancer 50 months later. Cancer of the gastric remnant was newly detected 36 months after distal gastrectomy. Death due to other diseases was observed in 4 patients (22.2%) without the recurrence of EGC: respiratory failure due to amyloidosis, hepatic failure due to cirrhosis, metastatic lung cancer detected after gastrectomy, and the recurrence of esophageal cancer treated by chemoradiotherapy before ESD.

A significant difference between Group A and Group B was observed in the age, tumor size, histologic differentiation, preoperative complications, surgical risk, and death by other diseases (Table 3). New disorders after surgery were frequently observed in Group A than Group B although this was not statistically significant. No case with gastric cancer recurrence was observed in Group A as well as Group B. Group A showed a less favorable outcome after surgery in terms of overall survival than Group B (*P* = 0.0087). However, the cancer survival rate after additional gastrectomy was the same in the two groups.

## 4. Discussion

There are several clinicopathologic issues in patients with submucosal cancer treated by additional gastrectomy after ESD for EGC. Two major points regarding diagnostic and therapeutic issues in these patients with additional gastrectomy were considered. One is a problem regarding EGC and metastasis, including incomplete resection by ESD, the presence of node metastasis before ESD, and the oversight of small EGC and metachronous cancer after ESD. The other is a problem regarding patients involving the surgical risk, other primary cancers, and new disorders after gastrectomy. On consideration of these issues, it is important that additional gastrectomy is employed based on the histopathologic findings after ESD.

There was only one case with a cancer remnant on the removed stomach although 9 showed positive lateral and/or vertical margins after ESD. Piecemeal resections of EGC by EMR have been reported to be associated with a high risk of local recurrence [1], and ESD for EGC has the advantage of being associated with a lower frequency of recurrence than EMR [2]. Furthermore, Yokoi et al. [15] reported that ESD facilitates the curative resection of locally recurrent EGC. In the present study, 94.4% of the cases with additional gastrectomy had no cancer remnant. This finding may be explained by burn degeneration at the cut-end of EGC treated by ESD. Tanabe et al. [16] reported that the mean width of burning degeneration at the cut ends of EGC treated by ESD was 1,203 *μ*m. Goto et al. [17] reported that preceding ESD for EGC had no negative influence on the prognosis when additional gastrectomy was performed, and it may be permissible to remove some EGC by ESD as a first step to prevent unnecessary gastrectomy. Therefore, cases without local recurrence of EGC may undergo the omission of additional gastrectomy when no node metastasis can be definitely shown.

Mutual features in the cases with node metastasis detected after additional gastrectomy were considered as follows: (1) a protruding type tumor, (2) over 25 mm in tumor size, (3) moderately differentiated type, (4) sm2, (5) positive lymphatic invasion, but (6) no cancer remnant after ESD. Furthermore, the 3 had preoperative comorbidities and a surgical history of laparotomy. Gotoda et al. [5] reported that 18.6% of submucosal EGC showed node metastasis histopathologically, while 91% of surgically treated EGC did not have node metastasis. Oda et al. [13] reported that 6.3% of noncurative patients with a possible risk of node metastasis after EMR/ESD for EGC showed regional node metastasis after gastrectomy. The present cases had to receive gastrectomy initially, because their histopathologic findings after ESD were considered to be associated with a high risk of node metastasis in EGC. However, they desired not to undergo surgery because of their advanced age, presence of physical and social complications, and history of laparotomy. It is hard to detect regional nodes with metastasis employing abdominal ultrasonography, endoscopic ultrasonography, CT, and conventional magnetic resonance imaging [18]. A new modality is necessary for an accurate diagnosis of node metastasis in patients with EGC before EMR/ESD and surgery. Additional gastrectomy based on the histopathologic findings after ESD is unnecessary in two thirds of the present cases without cancer remnants or node metastases, while the fact that additional gastrectomy is necessary in one third of the patients may be important. From the findings of the three cases with node metastasis detected after additional gastrectomy, we suggested that additional gastrectomy should be performed in EGC patients with co-morbidity showing over 25 mm in tumor size, sm2-invasion, and lymphatic invasion. However, it is possible that additional gastrectomy after ESD is avoided in the other patients, when no small cancer is detected endoscopically.

Regarding multiple gastric cancer after EMR/ESD, in the present study, 33.3% of the cases synchronously showed multiple EGC. Four lesions, detected after additional gastrectomy, were missed prior to ESD. Probably, endoscopists might overlook these lesions because of their small size. Nasu et al. [19] reported the characteristics of metachronous and synchronous EGC on initial EMR. Takenaka et al. [20] reported metachronous cancers of the gastric remnant after distal gastrectomy and the utility of ESD for EGC of the gastric remnant. Although EGC newly detected after additional gastrectomy was mucosal and small cancer, these lesions may have the potential to become metachronous cancer after ESD in the future.

Most of the patients undergoing additional gastrectomy hoped for treatment of EGC by ESD as a less-invasive procedure because they had several underlying diseases/preoperative comorbidities and surgical histories for other diseases. They were elderly and had a higher surgical risk in gastrectomy after ESD than routine gastrectomy for submucosal cancer. Furthermore, the nonelderly patients under 70 years old with additional gastrectomy also had several preoperative comorbidities. Hirasaki et al. [21] reported that elderly patients over 75 years old treated by ESD for EGC frequently had underlying diseases, but there were no differences in complications after ESD and the complete resection rate. Kakushima et al. [22] reported that the complete resection rate in ESD for EGC and the complication rate after ESD in elderly patients were not significantly different from those of younger patients. Because the number of elderly EGC patients with/without underlying diseases and surgical risk has been steadily increasing worldwide, we should pay attention to these EGC patients, treated by ESD and/or additional gastrectomy, regarding the clinicopathologic issues of age and comorbidities. Postoperative complications were frequently observed in the gastrectomized patients after ESD compared to those treated with routine gastrectomy; however, no mortality was observed in this series. No data from large-scale studies was found in postoperative morbidity and mortality comparing between EGC patients receiving additional and routine gastrectomy.

The disease free-survival rate after additional gastrectomy in patients with incomplete ESD for EGC was not worse [17], similar to the present study. No data were found for other primary cancers and new disorders, including mental/neurological disorders, in a long-term followup of EGC patients treated by additional gastrectomy after ESD. Approximately 1.5–5.4% of patients with subsequent cancer in other organs developing metachronously were detected after EGC treatment [23–25]. The number of patients with other subsequent cancers after surgery for EGC may not be so high, but a high frequency (16.7%) of other subsequent cancers was observed in the present study. Furthermore, no data were found regarding new disorders after additional gastrectomy for EGC, although the clinicopathologic studies of ESD in elderly patients with EGC were identified [21, 22]. The fact that the present cases with additional gastrectomy frequently had comorbidities/underlying diseases may affect the capacity to discover new disorders after additional gastrectomy. From these findings, it was considered that overall survival of cases with additional gastrectomy was poorer than in those receiving routine gastrectomy for submucosal EGC.

## 5. Conclusion

EGC patients with a number of comorbidities, including a surgical history and multiple cancers in other organs, may hope for less-invasive treatment by ESD. Consequently, additional gastrectomy may be recommended in one third, and we should consider several issues, including surgical problems as well as the complete resection of cancer and node metastasis before/after additional gastrectomy. Additional gastrectomy is safely performed in EGC patients with several comorbidities. It is possible that additional gastrectomy may be avoided in the other patients with comorbidities, when another small EGC may not be detected endoscopically. Regarding the followup, an issue that some of these patients died of other diseases remains. Furthermore, new modalities for diagnosis of lymph node metastasis may need for omission of additional gastrectomy.

## Figures and Tables

**Table 1:** Preoperative comorbidities in patients with additional gastrectomy.

Comorbidities	No. of cases
Cardiovascular diseases	10 (55.6%)
Hypertension	8
Angina pectoris	2
Complete atrioventricular block	1
Pericarditis induced by radiotherapy	1
Cerebral diseases	5 (27.8%)
Infarction	4
Hemorrhage	1
Diabetes mellitus	4 (22.2%)
Pulmonary diseases	4 (22.2%)
Chronic obstructive pulmonary disease	2
Interstitial pneumonia	2
Liver cirrhosis	2 (11.1%)
Dermatomyositis	2 (11.1%)
Idiopathic thrombocytopenic purpura	1 (5.6%)
Amyloidosis	1 (5.6%)

**Table 2:** Multiple cancers in other organs.

Other primary cancers	No. of cases	Treatment
Metachronous cancer before ESD	7	
Squamous cell carcinoma/esophagus	2	ESD (1), CRT (1)
Adenocarcinoma/duodenum	1	ESD
Adenocarcinoma/colon	2	Surgery
Hepatocellular carcinoma/liver	1	Surgery
Adenocarcinoma/ovary	1	Surgery
Squamous cell carcinoma/uterus	1	Surgery
Liposarcoma/retroperitoneum	1	Surgery
Synchronous cancer	1	
Hepatocellular carcinoma/liver	1	Surgery

ESD, endoscopic submucosal dissection; CRT, chemoradiotherapy.

**Table 3:** A Comparison of the clinicopathologic features of gastrectomized patients with and without ESD.

Variable		With ESD (*n* = 18)	Without ESD (*n* = 64)	*P* value
Age (mean ± SD: year-old)		72.5 ± 6.3	67.5 ± 10.8	0.029
Gender				0.39
	Men	15	49	
	Women	3	15	
Location				0.19
	Upper	9	19	
	Middle	6	22	
	Lower	3	23	
Tumor size (mean ± SD: mm		25.1 ± 12.1	35.1 ± 18.4	0.04
Gross type				0.08
	Protruding/elevated	11	21	
	Flat	0	2	
	Depressed/excavated	7	41	
Histologic differentiation				0.012
	Well/moderately	18	48	
	Poorly/signet ring cell	0	16	
Depth of invasion				0.71
	sm 1	4	17	
	sm 2	14	47	
Node metastasis				0.63
	Positive	3	11	
	Negative	15	53	
Lymphatic invasion				0.52
	Positive	8	34	
	Negative	10	30	
Venous invasion				0.057
	Positive	5	34	
	Negative	13	30	
Hepatic metastasis				0.78
	Positive	0	1	
	Negative	18	63	
Tumor number				0.19
	Solitary	12	52	
	Double or more	6	12	
Preoperative comorbidities				<0.001
	Positive	18	34	
	Negative	0	30	
History of major abdominal surgery				0.4
	Positive	6	15	
	Negative	12	49	
History of gastrectomy				0.7
	Positive	1	4	
	Negative	17	60	
History of major extra-abdominal surgery				0.38
	Positive	3	7	
	Negative	15	57	
Other primary cancer				0.3
	Positive	8	20	
	Negative	10	44	
Surgical risk				0.012
	Positive	18	48	
	Negative	0	16	
Operating time (mean ± SD: min)		312.5 ± 74.2	314.4 ± 82.5	0.77
	Total gastrectomy	327.2 ± 46.9	34.6 ± 65.0	0.57
	Distal gastrectomy	304.5 ± 86.7	293.8 ± 86.6	0.69
Operative blood loss (mean ± SD: mL)		190.6 ± 134.4	222.0 ± 148.0	0.43
	Total gastrectomy	206.7 ± 110.2	242.6 ± 166.4	0.79
	Distal gastrectomy	181.8 ± 150.3	208.9 ± 135.8	0.13
Postoperative complications				0.14
	Positive	9	20	
	Negative	9	44	
New disorders in followup				0.074
	Positive	9	19	
	Negative	9	45	
Death by other diseases				0.019
	Positive	4	2	
	Negative	14	62	

SD, standard deviation.
